# Global View of Ocular Parameter Changes Induced by a Single Hemodialysis Session

**DOI:** 10.3390/jcm15020592

**Published:** 2026-01-12

**Authors:** Joanna Roskal-Wałek, Joanna Gołębiewska, Jerzy Mackiewicz, Kamila Bołtuć-Dziugieł, Agnieszka Bociek, Paweł Wałek, Dominik Odrobina, Andrzej Jaroszyński

**Affiliations:** 1 Ophthalmology Clinic, Voivodeship Regional Hospital, 25-736 Kielce, Poland; 2 Collegium Medicum, Jan Kochanowski University, 25-516 Kielce, Poland; kbdziugiel@gmail.com (K.B.-D.); abociek28@gmail.com (A.B.); pawel.walek@o2.pl (P.W.); magdale_5@hotmail.com (D.O.); jaroszynskiaj@interia.pl (A.J.); 3 Department of Ophthalmology, Military Institute of Aviation Medicine, 01-755 Warsaw, Poland; 4 Medical Faculty, Lazarski University, 02-662 Warsaw, Poland; 5 Department of Vitreoretinal Surgery, Medical University of Lublin, 20-059 Lublin, Poland; jerzy.mackiewicz@umlub.pl; 6 1st Clinic of Cardiology and Electrotherapy, Swietokrzyskie Cardiology Centre, 25-736 Kielce, Poland; 7 Ophthalmology Clinic Boni Fratres Lodziensis, 93-357 Łódź, Poland

**Keywords:** end-stage renal disease, hemodialysis, ocular changes

## Abstract

**Background/Objectives:** Hemodialysis (HD) is the commonest life sustaining form of kidney replacement therapy in the world; however, this method of treatment have many adverse effects, and even a single HD session affects many organs, including the eyes. The aim of this study was to assess the effect of a single HD session on the ophthalmologic findings in patients with End-stage Renal Disease (ESRD). The second aim of the study was to examine the correlation of these changes with each other and between changes in systemic stressors related to the HD session. **Methods:** This was a single-center cross-sectional observational study conducted on 32 patients undergoing HD. Selected parameters of the anterior and posterior segment of the eye as well as systemic parameters were assessed before and after a single HD session. **Results:** Best corrected visual acuity (BCVA) improved, and lens thickness (LT), axial length (AXL), average macular thickness (MT), central MT and total vessel density (VD) of the deep capillary plexus DCP increased significantly after a single HD session. The Schirmer test results, tear break up time (TBUT), anterior chamber depth (ACD), central and average choroidal thickness (CT) decreased significantly after HD. Body weight loss was the only significant systemic change. Decrease in TBUT correlated positively with Schirmer’s test results decrease. Increase in CCT correlated positively with AXL increase. Decrease in central and average CT correlated positively with IOP decrease. Increase in central MT correlated positively with increase in average MT. Decrease in central CT correlated positively with average CT decrease. Change in VD of the SCP correlated positively with change in VD of DCP. Apart from the positive correlation between SBP change and Schirmer’s test results change, there were no correlations between systemic and ophthalmic parameters changes. **Conclusions:** Our study showed that HD affected the parameters of the anterior and posterior segments of the eye. Numerous correlations between these changes suggest that they are interrelated and represent the complex response of the eye to the HD process.

## 1. Introduction

End-stage renal disease (ESRD) is the final stage of chronic kidney disease (CKD), often necessitating such renal replacement therapy as dialysis or kidney transplantation [1]. The population of patients receiving dialysis continues to grow rapidly worldwide, with approximately 89% of patients on dialysis receiving hemodialysis (HD) [2]. HD is a life-sustaining therapy for ESRD patients; however, this method of treatment have many adverse effects, and even a single HD session affects many organs, including the eyes [3,4].

The growing number of studies are expanding our knowledge of the overall impact of HD on the visual system, indicating changes in intraocular pressure (IOP), central corneal thickness (CCT), and anterior chamber depth (ACD), along with alterations in the irido-corneal angle, lens thickness (LT), retinal thickness (RT), choroidal thickness (CT), and axial length (AXL), as well as vessel density (VD) changes. However, these findings present divergent results for almost all the assessed parameters [5,6,7,8,9,10,11,12,13,14,15,16,17,18,19,20]. Moreover, the relationships between the ocular changes resulting from an HD session have yet to be fully assessed.

Understanding the ocular changes resulting from HD sessions is crucial for several reasons. First, the awareness of the type and direction of these changes is important for the proper interpretation of the diagnostic test results and for selecting the most appropriate timing for the preoperative measurements and surgeries, especially cataract surgery [11,16]. Secondly, it is important to assess such changes in parameters as IOP, ACD, LT, and ocular blood flow in the context of the whether they lead to an increased risk of developing glaucomatous changes [8,10,12,13,16]. Furthermore, assessing the changes involving RT, CT, and VD provides a unique opportunity to assess the microcirculation status, which is the first to respond to the stress associated with HD. HD is associated with significant hemodynamic stress, which leads to challenged organ perfusion [3]. In this context, understanding the RT, CT, and VD changes has significant clinical implications. Nowadays, evaluating the ocular microcirculation seems promising as a potential biomarker for systemic microcirculation health [4,19].

The aim of this study was to investigate and analyze the impact of the HD session on various anterior and posterior segment parameters of the eye in individuals suffering from ESRD and to examine the correlation of these findings with each other and between systemic hemodynamic parameters.

## 2. Material and Methods

This prospective observational study was conducted between March 2022 and May 2022 at the Provincial General Hospital in Kielce. The study protocols were approved by the Bioethics Committee of the Jan Kochanowski University in Kielce (6/2022) and performed according to the provisions of the Declaration of Helsinki. Every participant provided written informed consent.

The study involved patients aged 18 years and above, of either gender, who had been diagnosed with ESRD and had currently been receiving HD treatment for at least three months. The inclusion criteria were the best corrected visual acuity (BCVA) exceeding 6/60 and no corneal pathology. Exclusion criteria were spherical equivalent refractive errors of more than +3.0 D and more than −5.0 D, an AXL of less than 21 mm or more than 26 mm, presence of pathological ocular conditions like media opacity that affects optical clarity, glaucoma, infection, and pathological conditions that could alter the structure of the retina or the choroid, such as macular degeneration, diabetic retinopathy, epiretinal membrane, macular edema, macular hole, and retinal vascular diseases. The exclusion criteria also included previous eye injury and history of ocular interventions like intravitreal injections, laser treatment, or eye surgery. Other exclusion criteria were atrial fibrillation, diagnosis of cerebral stroke or acute coronary syndrome within the previous 3 months, and the presence of severe infection or exacerbation of a chronic disease within 1 month before the study.

All patients were subjected to three 4 h HD sessions every week with a dialysate flow rate of at least 500 mL/min and a blood flow rate of at least 300 mL/min. Patients were examined on the same dialysis days (Mon/Wed/Fri). All the measurements were performed in the building housing the dialysis center. In order to eliminate the impact of diurnal variations, only patients subjected to morning HD sessions were included in the study.

Each patient underwent a full eye examination 30 min before HD (8 a.m.) and 15 min after HD, including a spherical equivalent (representative of refractive status) which was measured by an ophthalmologist using an automated refractometer (Righton Speedy-K2, UKON Corporation, Tokyo, Japan), a BCVA test measured on a logarithm of the minimum angle of resolution (logMAR) scale, IOP measured using a non-contact tonometer (Reichert7CR, AMETEK, Inc., Depew, NY, USA), and slit lamp examination of the anterior and posterior segments. CCT, ACD, LT, and AXL were evaluated using optical biometry (Tomey OA-2000 Optical Biometer, Tomey Corporation, Aichi, Japan).

Optical coherence tomography (OCT) and optical coherence tomography angiography (OCT-A) were obtained using swept-source OCT-A (DRI-OCT Triton SS-OCT Angio, Topcon Inc., Tokyo, Japan). The choroidal thickness (CT) and retinal thickness (RT) measurement was performed automatically in nine subfields of the ETDRS grid using 3D macula 7 × 7 mm scanning protocols. Average macular thickness (MT) and average CT were the average thickness of nine subfields of the ETDRS grid for each layer. Central MT and central CT refer to the central ETDRS subfield. The Vessel Density (VD) was calculated automatically by the software as the percentage area occupied by blood vessels in the three vascular plexi: Superficial Capillary Plexus (SCP), Deep Capillary Plexus (DCP), and Choriocapillaris (CC), using the ETDRS grid subfields to define the areas of interest. The VD was analyzed for each of the three vascular plexi in two predefined regions: (1) the central region, and (2) the entire 3 × 3 mm macular scan, referred to as total VD.

For the Schirmer test evaluation, a Schirmer paper of 5 mm width and 35 mm length with a bent end was inserted into the 1/3 lateral of the lower lid and removed after 5 min. The wetted length from the bent end was measured in mm and recorded.

For the tear break up time (TBUT) evaluation, sodium fluorescein strips were allowed to contact the lower fornix for a short period of time, and the cornea was stained through blinking. During the biomicroscopic examination, the patient was asked not to blink while a cobalt blue light was shone into the eye, and the time until the first black spots or lines formed in the tear layer on the cornea was recorded in seconds.

The body weight, Systolic Blood Pressure (SBP), Diastolic Blood Pressure (DBP), and Mean Arterial Pressure (MAP) were measured before and after HD. MAP was calculated using the following formula: DBP + 1/3 (SBP − DBP). The ultrafiltration volume represented the amount of fluid removed during HD and was recorded after HD.

Only one eye per participant was included in the analysis. The choice of the eye for the analysis was dictated by the better image quality of the OCT-A performed, on the basis of which such parameters as RT, CT, and VD were assessed. Scans with an image quality of at least 60% were considered eligible for analysis.

### Statistical Analysis

For each parameter, the differences between the values obtained before and after HD session were calculated. Next, the distributions of all variables were examined and, depending on their nature, the means and standard deviations or medians and interquartile ranges were calculated. Student’s *t*-test for dependent samples or the Wilcoxon signed-rank test were used to assess the significance of differences, depending on the distribution of the variables. The effect size (Cohen’s d coefficient for Student’s *t*-test) and Z/√N (for the Wilcoxon signed-rank test) were also calculated. To assess the relationship between changes in the values of individual parameters before and after HD, a correlation analysis was performed using Pearson’s r or Spearman’s rho, depending on the distribution of the studied characteristics. The level of statistical significance was set at *p* < 0.05. Analyses were performed using a STATISTICA 13.3 software (TIBCO Software Inc., Tulsa, OK, USA).

## 3. Results

A total of 48 patients were examined, of which 32 patients (14 males, 18 females; age 32–83 years; mean age 60.60 ± 12.33) met the inclusion criteria, and 32 eyes were included in the study. The etiology of ESRD included diabetes mellitus (*n* = 7), glomerulonephritis (*n* = 7), hypertension (*n* = 6), polycystic kidney disease (*n* = 4), vasculitis (*n* = 2), other (*n* = 3), and unknown (*n* = 3). Table 1 gives the baseline characteristics of the group.

### 3.1. Effect of Hemodialysis Session on Anterior Segment and Ocular Surface Parameters

Following a single HD session, BCVA improved (*p* = 0.05), ACD decreased (*p* = 0.003), while LT significantly increased (*p* = 0.039). IOP and CCT did not change. Regarding ocular surface changes, a single HD session resulted in a decrease in TBUT (*p* = 0.008) and Schirmer’s test results (*p* = 0.002). See Table 2 for more detailed changes in the anterior segment and ocular surface parameters.

### 3.2. Effect of Hemodialysis Session on Posterior Segment Parameters

Significant changes in the assessed posterior segment parameters included an increase in average and central MT (*p* = 0.0008 and *p* = 0.025, respectively) and a decrease in average and central CT (*p* = 0.0001). We did not demonstrate significant changes in VD in the SCP and in the CC. VD in total DCP increase (*p* = 0.03). See Table 3 for more about the detailed changes in the above-mentioned parameters.

### 3.3. Correlations Between Changes in Assessed Ophthalmologic Parameters

LT increase correlated negatively with ACD decrease. Decrease in TBUT correlated positively with Schirmer’s test results decrease. Change (increase) in CCT correlated positively with AXL increase. Decrease in central and average CT correlated positively with IOP decrease. Increase central MT correlated positively with increase in average MT, while decrease in central CT correlated positively with average CT decrease. See Table 4 for more detailed information about the correlations between the assessed ophthalmologic parameters.

### 3.4. Correlations Between Changes in Central VD in SCP, DCP, and CC and Changes in Central MT and CT

There was a significant positive correlation between changes in SCP VD and changes in DCP VD. No correlation was found between the VD changes in SCP, DCP, CC, and changes in central MT and CT. See Table 5 for more detailed information concerning the correlations between the assessed ophthalmologic parameters.

### 3.5. Correlation Between Changes in Assessed Ophthalmologicparameters and Systemic Parameters Changes

Positive correlation between the change in the SBP and the change in the Schirmer test results was the only correlation between assessed ophthalmologic parameters and systemic parameters changes (Table 6).

## 4. Discussion

Our study generated two major findings. First, an HD session was associated with significant changes in parameters of both the anterior and posterior segments of the eye. In this study, we found a significant reduction in ACD and an increase in LT. A single HD session was also associated with a significant reduction in the Schirmer test results and a shortening of TBUT. In the posterior segment, we observed a significant increase in AXL, central and average MT, VD of the total DCP, and a decrease in central and average CT. Second, we demonstrated numerous correlations between these changes, suggesting that they represent a complex response of the eye to the HD process.

HD typically lowers blood pressure and body weight and alters plasma protein concentrations [8]. During HD, toxic substances such as urea nitrogen and creatinine diffuse from the blood into the dialysate, causing a reduction in plasma osmotic pressure. Simultaneously, ultrafiltration gradually decreases extracellular fluid volume, which increases extracellular osmotic pressure. To compensate for this imbalance, water shifts from the intracellular to the extracellular compartment, leading to relative tissue dehydration. The eye consists largely of water in the form of aqueous humor and vitreous humor; therefore the fluids shifts during HD procedure may have a significant impact on the ocular parameters [10]. Changes in plasma osmolarity have been associated with alterations in ST, CCT, ACD, LT, IOP, AXL, RT, and CT observed after a single HD session [5,6,8,10,13,21]. Moreover, the choroid has the highest blood supply per area of any tissue and can therefore be a sensitive marker of hemodynamic changes generated during the HD session [4,5,8,10,22].

Most available studies have assessed only selected parameters of the anterior or posterior segment of the eye [5,6,7,8,9,10,11,12,13,14,15,16,17,18,19,20]. To the best of our knowledge, this is the first study to collectively present changes in so many ophthalmologic parameters associated with a single HD session and analyze their interrelationships. The variable results in the assessed ophthalmologic parameters of both the anterior and posterior segments of the eye presented in previous studies were due to methodological differences, including differences in the equipment and techniques used for measurements [5,6,7,8,9,10,11,12,13,14,15,16,17,18,19,20]. Techniques for assessing the anterior segment parameters have varied from ultrasound biomicroscopy and A-scan biometry to optical biometry, considered to be the most accurate method and used in the present study [5,6,7,8,9,10,11,12,13]. Similarly, different OCTA devices and scan protocols were used to assess the posterior segment parameters, which could have influenced the obtained results [14,15,16,17,18,19,20]. Also important are the differences between the study groups in terms of the etiology of ESRD, the time elapsed after HD before testing, and the extent of systemic changes resulting from HD [5,6,7,8,9,10,11,12,13,14,15,16,17,18,19,20]. However, each subsequent study provides increasingly detailed insight into ocular parameter changes due to a single HD session and is essential for understanding these complex mechanisms.

### 4.1. Anterior Segment Changes

#### 4.1.1. Central Corneal Thickness

Regarding the changes in the anterior segment, we noted no changes in CCT, similar to Chen et al. and Çalışkan et al. [5,11]. Chen et al. assessed also endothelial cell density, the average endothelial cell size, and the endothelial cell size variation coefficient, but these parameters, similarly to CCT, also did not change significantly [5]. However, it should be noted that endothelial cell density and morphology are important determinants of CCT stability and may influence CCT responses to HD sessions, which requires evaluation in further studies. Neither this nor previous studies have addressed the issue of corneal baseline status, which also should be considered in future research. In the study by Caglayan et al., the mean changes in CCT and corneal volume (CV) were −3.26 ± 7.03 µm and −0.90 ± 1.23 mm^3^, respectively (*p* = 0.002 and *p* < 0.001) [9]. Similarly, Kalayci et al. demonstrated a significant decrease in CCT [6]. Interestingly, in the study by Elbay et al., CCT did not show significant differences after HD; however, a significant increase in CCT was noted in the second hour of HD, suggesting a very dynamic but transient process that may result from fluid shifts due to changes in plasma osmolarity and oncotic pressure [8]. In our study, we found no correlation between changes in CCT and changes in systemic parameters. We did find a positive correlation between CCT increase and AXL increase.

It should be noted that changes in CCT may affect the values of other ophthalmologic parameters, such as IOP or ACD, which is the distance between the corneal epithelium and the lens [9].

#### 4.1.2. Anterior Chamber Depth

As a result of a single HD session, the ACD assessed in our study decreased significantly, similarly to the studies by Chen et al. and Lohokare et al. [5,13]. In other studies, an increase in ACD was visible [7], or ACD did not show significant changes [9,10,11].

Caglayan et al. assessed ACD, aqueous depth (AQD), anterior chamber volume (ACV), and anterior chamber angle (ACA) in the nasal and temporal quadrants, measured with a Sirius anterior segment analysis system. These parameters did not show significant changes due to HD [9].

In the study by Wang F. et al., changes in ACD were assessed depending on the anatomy of the iridocorneal angle. The study showed a non-significant decrease in central ACD after HD in all groups, ranging from 2.87 ± 0.31 to 2.84 ± 0.26 mm (*p* = 0.415) in the wide-angle group, from 2.69 ± 0.24 to 2.59 ± 0.27 mm (*p* = 0.051) in the narrow angle group, and from 2.65 ± 0.15 to 2.59 ± 0.16 mm (*p* = 0.338) in the extremely narrow-angle group [12].

It was assumed that the decrease in ACD could be attributed to total body fluid loss and increased plasma colloid osmotic pressure after HD, causing fluid efflux from the extravascular to the intravascular compartment [13]. In our study, we did not show any correlation between the change in ACD and systemic parameters.

In addition, the increased LT also contributes to a shallowing of ACD, as shown in our study, where a negative correlation was seen between a decrease in ACD and an increase in LT.

#### 4.1.3. Lens Thickness

The increase in LT seen in our study is consistent with the results of Wang L. et al. [10], where LT increased from 4.85 ± 0.41 mm to 4.90 ± 0.43 mm after HD, similar to the study by Lohokare et al. [13]. The researchers suggested that during HD, the concentration of urea nitrogen in the lens did not decrease as rapidly as in the blood, leading to an imbalance in the osmotic pressure between the lens and the aqueous humor. As a result, the lens absorbed water and dilated, resulting in an increase in LT [10]. Wang L. et al. notice that the increase in LT and forward positioning of the lens iris diaphragm may cause a shallow anterior chamber; however, the iris root and ciliary body in their study were both thinner, which should cause a widening of the anterior chamber. Therefore, these changes may overlap and ACD may not change significantly [10].

On the other hand, in the study by Chen et al., the mean LT decreased from 4.153 ± 0.413 mm to 4.056 ± 0.389 mm (*p* < 0.001). They hypothesized that the hemodialysis process partially dehydrated the lens, leading to a decrease in its mean thickness and an increase in its relative density. Although, the change in thickness was statistically significant but small, about 2% in lens thickness [5]. In the Çalışkan et al. study, mean LT did not change significantly during HD [11]. In our study, we found no correlation between LT change and the assessed systemic parameters changes.

#### 4.1.4. Intraocular Pressure

One parameter that has been assessed in many studies, and there is still no consensus on the direction of changes during HD sessions, is IOP [5,6,7,8,9,10,11,12,13,14,15]. Our study, similarly, to Chen et al., Wang L. et al., and Çalışkan et al., found no significant difference in IOP following a single HD session [5,10,11]. Some authors indicate a decrease in IOP [6,7,9].

In the study by Elbay et al., mean intraocular pressure increased significantly, from 16.20 ± 3.27 mmHg before hemodialysis to 18.28 ± 3.79 mmHg in the second hour of hemodialysis (*p* = 0.001). After HD, it decreased to 16.09 ± 2.97 mmHg, which was not significantly different from the pre-HD value (*p* = 0.844). These results support the importance of monitoring intraocular pressure during HD, especially in patients with glaucoma [8].

It is hypothesized that, due to the presence of the blood–ocular barrier, the decrease in serum osmolarity induced by HD leads to an osmotic gradient between the plasma and intraocular fluids, which in turn leads to the movement of water from the plasma into the eye. If the aqueous humor outflow pathway is not obstructed, this increased amount of aqueous humor is drained from the eye, and the intraocular pressure remains normal. If the aqueous humor outflow is impaired, the IOP increases [21].

The influence of the morphology of the iridocorneal angle on IOP changes following HD sessions was assessed in the study by Wang F. et al. The authors showed that the IOP of the extremely narrow-angle group increased significantly after HD (from 17.0 ± 4.7 to 18.4 ± 3.8 mmHg) (*p* < 0.05). The authors indicate that an extremely narrow angle is a risk factor for elevated IOP during HD, while wide-angle patients are relatively safe [12].

In the study by Lohokare et al., the change in IOP was not statistically significant (*p* = 0.455). However, during the study period, eight patients developed acute angle-closure glaucoma after the HD sessions. The authors note that in such cases, it would be beneficial to consider prophylactic laser iridotomy before the HD session [13]. In our study, we excluded patients with glaucoma, patients with an AXL of less than 21 mm, and hyperopia, which most likely accounts for the lack of cases of acute angle closure in our group.

Patients with chronic renal failure have insufficiency in the local blood supply to the eyes. Induction by HD session fluctuations in the IOP and shallowing of the ACD may worsen this condition or even result in severe irreversible ischemic and hypoxic damage to the optic nerve and retina [12].

In the study by Chen et al., where HD resulted in a visible but not significant increase in IOP, a negative correlation was observed between changes in IOP and diastolic pressure [5].

Although we did not find significant changes in IOP, this small change in the direction of IOP decrease correlated with a decrease in CT. In the study by Wang L. et al., the iris root and ciliary body were both thinner, while a decrease in CT was also demonstrated. These changes may influence IOP both through positioning of the iris–lens diaphragm and through aqueous humor production and outflow. However, this study did not demonstrate a correlation between the decrease in CCT and the change in IOP [10]. In the study by Kalayci et al., a significant correlation was observed between change in AXL and change in IOP (r = 0.202, *p* = 0.03) [6].

#### 4.1.5. Axial Length

In the study by Kalayci et al., the mean AXL decreased from 23.1 ± 0.8 mm to 22.9 ± 0.8 mm, and the difference was significant (*p* < 0.001) [6].

Wang L. et al. observed a significant increase in the vitreous axial length (VAL) from 15.43 ± 0.82 mm to 15.48 ± 0.82 mm (*p* < 0.01) and the AXL from 23.04 ± 0.79 mm to 23.10 ± 0.79 mm (*p* < 0.01) after HD. The authors assumed that decrease in plasma osmotic pressure, accompanied by a simultaneous decrease in osmotic pressure in the structures of the eyeball, promoted the movement of water into the vitreous, resulting in its elongation. As a result of changes in LT and VAL, the total AXL of the eye increased after HD [10].

For Ismayılov et al., AXL was measured using ultrasound biometry and showed a significant increase from 23.05 ± 1.35 to 23.13 ± 1.35 mm (*p* < 0.001) [7]. Similarly to the Çalışkan et al. study, which used optical biometry to measure AXL, we found a significant increase in AXL [11]. In the Çalışkan et al. study, although the change in AXL was statistically significant, it had a small effect size (Cohen’s d = 0.06), which reduced its clinical significance. In our case, the effect size was definitely higher (Cohen’s d = 0.50). In the Çalışkan et al. study, no significant changes were observed in the parameters of the anterior part of the eye (including CCT and ACD), which could have influenced the obtained results. The authors hypothesized that the increase in AXL could have resulted from posterior displacement of the internal limiting membrane caused by a decrease in retinal and/or choroidal thickness [11]. In our study, significant changes in MT and CT were not correlated with the change in AXL; more importantly, they were opposite. We demonstrated a positive correlation between AXL increase and CCT increase.

Accurate ocular biometric measurements are essential for acceptable outcomes in cataract and refractive surgery. AXL and corneal keratometry are the two main determining factors in IOL power calculation. In the study by Çalışkan et al., keratometry readings were unchanged. Although AXL increased significantly after HD, the HD session did not significantly affect intraocular lens (IOL) power calculations [11]. However, this result requires verification in subsequent studies due to the small effect size of the AXL change. In our study, the change in AXL was associated with a large effect size; however, due to the lack of keratometry measurements, we were unable to assess the impact of these key variables on IOL power calculations.

### 4.2. Ocular Surface

The results regarding the effect of HD on the ocular surface are more consistent and indicate that HD reduces tear secretion with a decrease in the Schirmer test and a shortening of TBUT, which was evident in our study. Decrease in TBUT correlated positively with a Schirmer’s test results decrease. Increased tear hyperosmolarity resulting from elevated tear urea levels induces inflammatory cytokines and matrix metalloproteases on the surface of the epithelial cells. This process leads to ocular surface damage, loss of goblet cells, decreased mucin expression, and tear instability [6,7].

The study by Kalayci et al. also showed a decrease in TBUT and Schirmer’s test. In the correlation analysis, a significant association was found between BST and plasma colloid osmotic pressure, and between serum osmolarity and the keratoepitheliopathy score [6].

In our study, the decrease in ST results correlated positively with the decrease in SBP, which may be related to dehydration resulting from the HD session. The observed changes may also be due to the autonomic nervous system (ANS) response, but this requires evaluation in future studies.

### 4.3. Posterior Segment Changes

#### 4.3.1. OCT Structural Parameters

In our study, we found significant changes in MT and CT. MT increased significantly, while CT decreased. Similar results were presented by Wang et al. [10]. The observed changes may result from different mechanisms of blood flow regulation in the retina and choroid. Retinal circulation is autoregulated and is compared to cerebral circulation, while choroidal circulation is mainly controlled by extrinsic autonomic innervation. Moreover, it should be borne in mind that the retina is a part of the nervous system [10,22]. It was demonstrated that within a single dialysis session, there is acute development of new cytotoxic edema of the brains of patients receiving HD [3]. In the study by Chen et al., RT tended to increase in different locations and different layers. The authors suggested that HD reduces the plasma crystal osmotic pressure such that the liquid goes into the layers of the retina along the concentration gradient, thickening the retina and leading to edema [5]. Aquaporins (AQPs), a family of transmembrane water channel proteins, play a significant role in regulating water homeostasis across various tissues, including the nervous system. They are crucial for maintaining water balance and for the development and resolution of edema [23].

We suppose that the type of AQP and the degree of its expression are related to the increase in MT in this and in our previous study, but this assumption requires evaluation in subsequent studies [16]. Moreover, we can only speculate that the changes in AQP channels in the lens, cornea, and ciliary epithelium are also involved in the observed changes in IOP, CCT, and LT.

#### 4.3.2. OCTA Perfusion Metrics

It remains unclear whether the changes in the vascular compartment influence the increase in RT after a single HD session. Lin et al. found an increase in RT after the HD session and suggested that increase in VD in DCP may be involved in this change. Similarly to Lin et al., we found significant increase in RT and significant increase in VD in the DCP. The authors suggested that the significant increase in VD in DCP may be caused by varying degrees of vasodilation in retinal vessels [17].

In other studies the lack of significant changes in SCP and DCP following a single HD session was related to retinal blood flow autoregulation [15,18]. Shin et al. observed no significant associations between the changes in VD in the SCP and DCP, and the systemic factors, such as the changes in SBP, DBP, MAP, and ultrafiltration rate. However, in this study, the reduction in the CC VD was associated with the BP decrease, including SBP and MAP. The decrease in CT also correlated with the ultrafiltration volume [18]. These results may suggest that choroid and choroidal circulation could be a sensitive marker of hemodynamic changes induced by the HD session.

#### 4.3.3. Choroidal Metrics

Coppolino et al. found a decrease in VD in the retinal plexuses during the HD session but noted that retinal parameters smoothly adapted to fluid removal, conversely to CT, which decreased from the start to the end of HD and achieving a marked significance [19]. Similarly, in our study we observed a significant decrease in the CT. Most of the studies show a CT reduction after HD, and in some of them the decrease in CT was accompanied by a CC VD reduction [4,24]. In our study, we did not demonstrate an association between the change in CC VD and the change in central CT.

Li et al. used wide-field OCTA to assess the Perfusion Area (PA) and Choroidal Vascularity Index (CVI) to evaluate the alterations in choroidal blood flow post HD, which was expected to mirror the systemic blood flow changes. Both of these parameters decreased after HD in several quadrants. The decline in CVI in the optical disk area after HD was more evident compared to the macular region. The authors suggest that alterations in blood flow within the optic disk and choroidal layers are more susceptible and therefore may serve as early indicators of systemic hemodynamic changes [20].

In our study, the change in CT did not correlate with any of the assessed systemic parameters, i.e., SBP, DBP, MAP, and body weight change. It was noted that the lack of choroidal autoregulation may contribute to the observed changes in CT. Some authors attribute the reduction in CT following HD sessions to autonomic nervous system (ANS) function [4,14]. ANS plays a key role in maintaining hemodynamic stability [25]. The parasympathetic innervation has been shown to vasodilate and increase choroidal blood flow, and the sympathetic input has been shown to vasoconstrict and decrease choroidal blood flow [26]. It is assumed that sympathetic activation induced by blood volume depletion during HD causes stronger contraction of vascular smooth muscle and nonvascular smooth muscle in the choroid, which consequently leads to a decrease in CT [4,14]. This is a very interesting assumption, but it requires verification in further research.

Interestingly, the choroid plexus is the only non-autoregulated vascular bed in the body [3]. Several studies reported that the choroidal circulation is not autoregulated. However, other authors found some evidence for autoregulation in the choroidal vasculature [22,26]. Hemodialysis results in a significant reduction in the choroid plexus perfusion pressure and an acute intradialytic reduction in the cerebrospinal fluid, which is produced by the choroid plexus [3]. Similarly, we observed a significant CT reduction after the HD session, with a positive correlation with changes in IOP, which also was reduced—however, this reduction was not significant.

Changes in the CCT, LT, RT, and CT, as well as changes in AXL, which may occur as a result of HD, can affect visual acuity. In our study, we demonstrated improvement in BCVA. However, in the studies by Sun et al. and Chen et al., BCVA did not change significantly [5,14]. Similarly to the study by Sun et al., in our study the BCVA changes were insignificantly correlated with any systemic parameters [14].

### 4.4. Strength

This is the first study to analyze both anterior and posterior segment parameters with OCT-A parameters. The study was conducted at a single center just before and after HD, significantly eliminating the influence of time on the regression of changes resulting from HD. We also present the impact of stressors (changes in systemic parameters associated with HD) and their impact on the parameters assessed in the study.

### 4.5. Limitation

The first major limitation of the study is the relatively small study group. Moreover, due to the small sample size, we did not assess the impact of HD on the different causes of ESRD. Due to the study’s exclusion criteria regarding retinal pathology, the DM-ESRD group could not be thoroughly analyzed. Consequently, the generalizability of our findings to the broader DM-ESRD population may be limited.

The second limitation is the difference in timing of the systemic and ocular measurements. The systemic parameters were measured immediately before and after HD, whereas ocular parameters were measured 30 min before and 15 min after HD. During this time, the patients were transferred to another room for ophthalmologic examination, and it is unclear if the systemic parameters changed after this period.

The third limitation is that the ocular parameters were measured only before and after a single HD session. We did not assess selected parameters in subsequent hours and days, and we do not know how quickly these parameters change or when and if they return to their baseline values.

The fourth limitation is that we did not have patients with glaucoma, and we did not observe IOP elevations or angle closure, as occurred in other studies. We did not perform gonioscopy, nor did we assess the iridocorneal angle or changes in its range.

The fifth limitation is the absence of keratometry readings, which prevented assessment of the HD session’s effect on IOL power calculation.

The sixth limitation is the lack of specular microscopy, precluding the assessment of endothelial cell density and morphology.

The seventh limitation is that we did not consider ANS function and the influence of drugs, changes in gas concentration, pH, or changes in Na, K, and Ca concentrations on the assessed parameters.

## 5. Conclusions

Our study showed that HD affected the parameters of the anterior and posterior segments of the eye. Numerous correlations between these ophthalmologic changes suggest that they are interrelated and represent the complex response of the eye to the HD process. The significant changes observed in selected ophthalmologic parameters, such as AXL and ACD, raise the question of the impact of HD sessions on IOL power calculation—an issue that should be addressed in future studies. It should also be noted that recurrent changes following HD sessions (e.g., increased RT and decreased CT) may be responsible for the subsequent decline in CT, RT, and VD values. However, this assumption also requires verification in future studies.

## Figures and Tables

**Table 1:** Baseline characteristics of the study group.

Patients’ Characteristic	Before HD	After HD	*p*
Age (years) Mean (SD)	60.60 ± 12.33		
Gender Females (%) Males (%)	18 (56.25) 14 (43.75%)		
Duration of HD (years) Mean (SD)	4.70 ± 3.56		
Ultrafiltration volume (L) Mean (SD)	2.19 ± 1.05		
kt/v Mean (SD)	1.61 ± 0.31		
Spherical equivalent (D) Mean (SD)	0.17 ± 1.04		
Body weight (kg)	76.95 ± 18.01	74.76 ± 17.49	<0.0001 ^A^
SBP (mmHg)	143.13 ± 26.97	140.61 ± 27.87	0.6525 ^A^
DBP (mmHg)	78.66 ± 13.68	80.06 ± 13.40	0.4717 ^A^
MAP (mmHg)	100.15 ± 17.15	100.25 ± 17.23	0.8916 ^A^

Abbreviations: DBP—Diastolic Blood Pressure; HD—Hemodialysis; MAP—Mean Arterial Pressure; SBP—Systolic Blood Pressure; SD—Standard Deviation; ^A^—Student’s *t* test for dependent variables.

**Table 2:** Effect of hemodialysis session on anterior segment and ocular surface parameters.

Variables	Before HD	After HD	*p*	EF
**BCVA, (logMAR)**	0.12; 0.21 ^B^	0.00; 0.24 ^B^	0.0504 ^W^	0.35 ^r^
**IOP (mmHg)**	15.0; 5.5 ^B^	15.0; 3.50 ^B^	0.804 ^W^	0.05 ^r^
**CCT (µm)**	536.78 ± 31.04 ^A^	538.04 ± 31.37 ^A^	0.311 ^T^	0.03 ^r2^
**ACD (mm)**	3.17; 0.62 ^B^	3.14; 0.71 ^B^	0.003 ^W^	0.57 ^r^
**LT (mm)**	4.80 ± 0.43 ^A^	4.82 ± 0.44 ^A^	0.039 ^T^	0.36 ^r2^
**Schirmer test (mm)**	25.0; 16 ^B^	20.5; 17.0 ^B^	0.002 ^W^	0.71 ^r^
**TBUT (s)**	11.75; 7.0 ^B^	8.0; 7.5 ^B^	0.008 ^W^	0.47 ^r^

Abbreviations: ACD—Anterior Chamber Depth; BCVA—Best Corrected Visual Acuity; CCT—Central Corneal Thickness; IOP—Intraocular Pressure; LT—Lens Thickness; TBUT—Tear Break-Up Time. ^A^—M—Mean; ^B^—Me—Median; ^T^—Test t for dependent variables; ^W^—Wilcoxon rank test. Statistically significant difference (*p* < 0.05) ^r^ Effect size for Wilcoxon rank test: r = Z/$\sqrt{N};$ small effect: 0.10 to <0.30; moderate effect: 0.30 to <0.50; large effect: ≥0.50; effect size for *t* test for dependent variables: r^2^ = $\frac{t^{2}}{t^{2}+df}$; r^2^ = 0.01—small effect; r^2^ = 0.09—medium effect; r^2^ = 0.25—large effect.

**Table 3:** Effect of hemodialysis session on posterior segment parameters.

Variables	Before HD	After HD	*p*	EF
**AXL (mm)**	22.90 ± 0.77 ^A^	22.92 ± 0.77 ^A^	0.001 ^T^	0.50 ^r2^
**Central MT (μm)**	236.13 ± 27.76 ^A^	240.47 ± 25.64 ^A^	0.025 ^T^	0.15 ^r2^
**Average MT (μm)**	271.06; 20.83 ^B^	274.06; 19.28 ^B^	0.0008 ^W^	0.59 ^r^
**Central CT (μm)**	233.03 ± 73.85 ^A^	219.22 ± 70.78 ^A^	0.0001 ^T^	0.58 ^r2^
**Average CT (μm)**	223.34 ± 67.49 ^A^	210.38 ± 67.44 ^A^	0.0001 ^T^	0.54 ^r2^
**Central SCP VD**	20.46 ± 3.99 ^A^	20.84 ± 4.06 ^A^	0.446 ^T^	0.02 ^r2^
**Total SCP VD**	40.8; 2.46 ^B^	41.28; 2.93 ^B^	0.3037 ^W^	0.18 ^r^
**Central DCP VD**	18.12 ± 4.20 ^A^	19.16 ± 4.58 ^A^	0.1663 ^T^	0.07 ^r2^
**Total DCP VD**	39.99; 3.25 ^B^	40.54; 2.98 ^B^	0.0300 ^W^	0.38 ^r^
**Central CC VD**	50.80; 5.20 ^B^	52.80; 4.76 ^B^	0.0787 ^W^	0.31 ^r^
**Total CC VD**	52.70; 2.03 ^B^	52.93; 1.49 ^B^	0.0998 ^W^	0.29 ^r^

Abbreviations: AXL—Axial Length; CC—Choriocapillaris; CT—Choroidal Thickness; DCP—Deep Capillary Plexus; HD—Hemodialysis; MT—Macular Thickness; SCP—Superficial Capillary Plexus; VD—Vessel Density; ^T^—Student test T for dependent variables; ^W^—Wilcoxon rank test; ^A^—M—Mean; ^B^ Me—Median; ^r^ Effect size for Wilcoxon rank test: r = Z/√(N;) small effect: 0.10 to <0.30; moderate effect: 0.30 to <0.50; large effect: ≥0.50; effect size for *t* test for dependent variables: r^2^ = t^2/(t^2 + df); r^2^ = 0.01—small effect; r^2^ = 0.09—medium effect; r^2^ = 0.25—large effect; Statistically significant difference (*p* < 0.05).

**Table 4:** Correlations between changes in assessed ophthalmologic parameters.

Variables	ΔBCVA	Δ IOP	Δ CCT	Δ ACD	Δ LT	Δ AXL	Δ Central MT	Δ Average MT	Δ Central CT	Δ Average CT	Δ ST	Δ TBUT
Δ BCVA												
Δ IOP	−0.02 ^S^											
Δ CCT	0.11 ^S^	0.12 ^S^										
Δ ACD	0.16 ^S^	0.19 ^S^	0.09 ^S^									
Δ LT	−0.19 ^S^	−0.26 ^S^	−0.09 ^S^	−0.46 ^(0.008) S^								
Δ AXL	0.17 ^S^	0.01 ^S^	**0.35** **^(0.04) S^**	−0.13 ^S^	0.20 ^S^							
Δ central MT	0.24 ^S^	−0.14 ^S^	0.26 ^S^	0.16 ^S^	−0.07 ^S^	−0.04 ^S^						
Δ average MT	0.32 **^S^**	−0.21 ^S^	0.18 ^S^	0.02 ^S^	−0.03 ^S^	0.02 ^S^	0.77 ^(0.001) S^					
Δ central CT	−0.11 ^S^	0.45 ^(0.009) P^	−0.02 ^S^	0.29 ^T^	−0.01 ^S^	−0.31 ^P^	−0.18 ^S^	−0.17 ^S^				
Δ average CT	−0.13 ^S^	0.41 ^(0.009) P^	−0.15 ^S^	0.34 ^T^	−0.04 ^S^	−0.24 ^P^	−0.31 ^S^	−0.32 ^S^	0.68 ^(0.001) P^			
Δ ST	0.00 ^S^	0.18 ^S^	0.15 ^S^	−0.01 ^S^	0.15 ^S^	0.03 ^S^	−0.09 ^S^	−0.09 ^S^	0.09 ^S^	0.19 ^S^		
Δ TBUT	−0.21 ^S^	0.19 ^P^	0.25 ^S^	0.05 ^P^	0.11 ^S^	−0.26 ^P^	0.25 ^S^	0.15 ^S^	0.17 ^P^	−0.03 ^P^	0.42 ^(0.016) S^	

Abbreviations: ACD—Anterior Chamber Depth; BCVA—Best Corrected Visual Acuity; CCT—Central Corneal Thickness; CT—Choroidal Thickness; IOP—Intraocular Pressure; LT—Lens Thickness; TBUT—Tear Break-Up Time; MT—Macular Thickness; ST—Schirmer test; ^S^—Spearman rank correlation; ^P^—Pearson correlation. ^T^—Student test T for dependent variables.

**Table 5:** Correlations between changes in central VD in SCP, DCP, and CC and changes in central MT and CT.

Variables	Δ Central MT	Δ Central CT	Δ Central SCP VD	Δ Central DCP VD	Δ Central CC VD
Δ central MT					
Δ central CT	−0.18 ^S^				
Δ Central SCP VD	−0.01 ^S^	−0.16 ^P^			
Δ Central DCP VD	0.19 ^S^	−0.08 ^P^	0.83 ^(0.001) P^		
Δ Central CC VD	−0.22 ^S^	−0.24 ^S^	0.26 ^S^	0.09 ^S^	

Abbreviations: CC—Choriocapillaris; CT—Choroidal Thickness; DCP—Deep Capillary Plexus; MT—Macular Thickness; SCP—Superficial Capillary Plexus; VD—Vessel Density; ^S^—Spearman rank correlation; ^P^—Pearson correlation.

**Table 6:** Correlations between changes in the assessed ophthalmologic parameters and the systemic parameters.

Variables	Δ SBP		Δ DBP		Δ MAP		Δ Body Weight	
	R	*p*	R	*p*	R	*p*	R	*p*
Δ BCVA	−0.16	0.386 ^S^	−0.13	0.474 ^S^	−0.13	0.481 ^S^	−0.11	0.564 ^S^
Δ IOP	0.17	0.368 ^P^	0.22	0.244 ^P^	0.20	0.280 ^P^	−0.09	0.639 ^P^
Δ CCT	0.27	0.146 ^S^	0.13	0.496 ^S^	0.21	0.251 ^S^	0.07	0.709 ^S^
Δ ACD	−0.09	0.645 ^S^	−0.17	0.368 ^S^	−0.13	0.478 ^S^	0.07	0.721 ^S^
Δ LT	−0.02	0.910 ^S^	−0.03	0.864 ^S^	−0.04	0.840 ^S^	−0.02	0.914 ^S^
Δ AXL	0.12	0.512 ^P^	0.11	0.542 ^P^	0.12	0.508 ^P^	−0.19	0.311 ^P^
Δ central MT	−0.15	0.423 ^S^	−0.20	0.248 ^S^	−0.20	0.293 ^S^	−0.08	0.672 ^S^
Δ Average MT	−0.11	0.571 ^S^	−0.21	0.251 ^S^	−0.18	0.336 ^S^	−0.22	0.227 ^S^
Δ Central CT	0.11	0.564 ^P^	0.05	0.807 ^P^	0.08	0.666 ^P^	−0.03	0.892 ^P^
Δ Average CT	0.18	0.343 ^P^	0.20	0.289 ^P^	0.20	0.293 ^P^	0.18	0.344 ^P^
Δ Central SCP VD	0.04	0.840 ^P^	0.04	0.775 ^P^	−0.02	0.923 ^P^	−0.06	0.753 ^P^
Δ Total SCP VD	0.04	0.840 ^P^	0.04	0.811 ^P^	0.084	0.733 ^P^	−0.06	0.753 ^P^
Δ Central DCP VD	0.078	0.713 ^P^	0.10	0.604 ^P^	0.609	0.643 ^P^	−0.17	0.370 ^P^
Δ Total DCP VD	0.19	0.313 ^P^	0.08	0.652 ^P^	0.13	0.482 ^P^	−0.07	0.686 ^P^
Δ Central CC VD	−0.28	0.130 ^S^	−0.21	0.257 ^S^	−0.28	0.121 ^S^	−0.11	0.555 ^S^
Δ Total CC VD	−0.10	0.584 ^P^	0.03	0.871 ^P^	−0.04	0.838 ^P^	−0.02	0.931 ^P^
Δ ST	0.38	0.033 ^S^	0.07	0.709 ^S^	0.21	0.248 ^S^	−0.04	0.844 ^S^
Δ TBUT	0.30	0.104 ^P^	0.19	0.299 ^P^	0.26	0.162 ^P^	−0.01	0.976 ^P^

Abbreviations: ACD—Anterior Chamber depth; BCVA—Best Corrected Visual Acuity; CC—Choriocapillaris; CCT—Central Corneal thickness; CT—Choroidal Thickness; DBP—Diastolic Blood Pressure; DCP—Deep Capillary Plexus; IOP—Intraocular Pressure; LT—Lens Thickness; MAP—Mean Arterial Pressure; MT—Macular Thickness; SBP—Systolic Blood Pressure; SCP—Superficial Capillary Plexus; ST—Schirmer test; TBUT—Tear Break-Up Time; VD—Vessel Density. ^S^—Spearman rank correlation; ^P^—Pearson correlation.

## Data Availability

The original contributions presented in this study are included in the article. Further inquiries can be directed to the corresponding authors.
